# National Norms and Differential Item Functioning Tests of the Parent-Report MFQ for Children Ages 5–12

**DOI:** 10.1007/s10862-026-10300-9

**Published:** 2026-07-17

**Authors:** Rachel A. Vaughn-Coaxum, Krithika Prakash, Claire S. Tomlinson, Patrick Mair, Lan Yu, David A. Kolko, Paul A. Pilkonis, Oliver Lindhiem

**Affiliations:** 1 Department of Psychiatry, University of Pittsburgh, School of Medicine, 100 N. Bellefield Ave, Pittsburgh, PA 15213 USA; 2 Department of Psychology, Harvard University, Cambridge, MA USA; 3 Department of Medicine, University of Pittsburgh, School of Medicine, Pittsburgh, PA 15213 USA; 4 University of Pittsburgh Medical Center, Western Psychiatric Institute and Clinic, Pittsburgh, PA USA

**Keywords:** Depression, Mood and feelings questionnaire, Item response theory, parent report, Nationally representative

## Abstract

The Mood and Feelings Questionnaire (MFQ) is a widely used screening tool for childhood depression, but existing cutoff scores have been derived primarily from older or clinically referred samples of youth. Less is known about score distributions and percentiles in nationally representative samples of younger children. The present study established parent-report MFQ norms and evaluated item characteristics and Differential Item Functioning (DIF) across parent race/ethnicity, and parent and child sex, in a large, population-based sample. Data were collected in February 2024 from 1,000 U.S. parents of children ages 5–12. Parents completed demographic measures and the 34-item long-form MFQ. Item response theory (IRT) analyses evaluated unidimensionality, item discrimination, and DIF across the three largest racial/ethnic groups (White, Black, Latine) and across parent and child sex. Percentile ranks and 95% confidence intervals were generated for MFQ raw total scores. Results supported unidimensionality and good model fit. All items demonstrated adequate to strong discrimination, and no items showed meaningful DIF across subgroups. Percentile norms indicated that a raw score of 15 corresponded to the 85th percentile, providing a developmentally appropriate cutoff for younger children in community samples. These findings augment the evidence base for the MFQ by providing nationally representative norms for younger children, with practical implications for clinical interpretation of total scale scores.

**Supplementary Information:**

The online version contains supplementary material available at https://doi.org/10.1007/s10862-026-10300-9.

The assessment of depression symptoms in children has largely relied on self-report and caregiver report questionnaires to identify the presence and severity of symptoms. There is a wide range of published and validated measures of youth depression symptoms spanning childhood and adolescence (Roseman et al., 2016). Clinically, youth self-report and caregiver-report of youths’ symptoms are used for screening, symptom monitoring, and assessment of treatment outcomes (i.e., measurement-based care). In research, these measures are used to evaluate the effectiveness of interventions, measure the impact of risk factors on the etiology of depression symptoms, and determine who is included or excluded from experimental studies and clinical trials based on the presence or severity of symptoms. Many of the most widely used self-report depression symptom inventories for youth (e.g., Children’s Depression Inventory [CDI], Center for Epidemiological Studies Depression Scale for Children [CESD], Mood and Feelings Questionnaire [MFQ], Beck Depression Inventory [BDI]) were originally developed in the 1980s and 1990s (Costello & Angold, 1988), with much of the psychometric data cited for their use also originating within the decade following publication.

The Mood and Feelings Questionnaire (MFQ; Angold et al., 1996) is one such measure that is widely used clinically and in psychological and psychiatric research. A search of clinical trials with youth (17 years of age and under) registered with the U.S. federal government (clinicaltrials.gov) indicates that 316 clinical trials specified the MFQ as an outcome measure between 2005 and 2025, and 270 trials include youth under the age of 13. Originally developed in 1986, with a short form published in 1995, psychometric evaluations of the MFQ were originally published in the late 1990s (Kent et al., 1997; Thapar & McGuffin, 1998; Wood et al., 1995) with population-specific evaluations (e.g., youth in New Zealand, Spanish translations, Arabic translations; Fernández-Martínez et al., 2020; Noureddine et al., 2025; Sharp et al., 2006; Thabrew et al., 2018) and setting-specific evaluations (e.g., inpatient vs. outpatient, Burleson Daviss et al., 2006; Jeffreys et al., 2016) through the 2000s. These studies included initial tests of reliability and validity. Three later studies used item response theory (IRT; Sharp et al., 2006; Wang & Gao, 2025) approaches to evaluate item-level performance of the youth-report MFQ and to examine differential item functioning (DIF; Banh et al., 2012) across youth’s racial and ethnic identities. Another study used confirmatory factor analysis to evaluate measurement invariance across youth development and sex (Schlechter et al., 2023). More recent psychometric evaluation of the parent-report version of the MFQ has been limited to evaluating its factor structure (Fernández-Martínez et al., 2020; Jeffreys et al., 2016; Noureddine et al., 2025). However, studies of item performance and DIF evaluation are limited for the parent-report MFQ in the psychometric literature to date.

Continued evaluation of measure performance and assessment of DIF is critical for interpreting scores from instruments that assess youth psychopathology. There are potential cohort effects that may influence the performance of questionnaire items assessing youth depression symptoms, and the demographic characteristics of the youth population in the United States have changed substantially since the publication of the MFQ and similar depression instruments in the 1990s. Importantly, samples of youth included in the early psychometric publications may have been representative of the population at the time, but may not be population representative at present (Federal Interagency Forum on Child and Family Statistics, 2023; US Census Bureau, 2022, 2022): For example, youth identifying racially as non-Hispanic White have decreased from 68.9% of U.S. children under 18 at the time of the MFQ publication to 48.9% in 2022. Youth identifying as Hispanic or Latine have increased from 14.9% to 26% of U.S. children under 18, while youth racially identifying from the Asian and Pacific Island diasporas have increased from 3.4 to 6.2% of U.S. children under 18.

Assessing DIF in self- and parent-report measures of youth depression symptoms is critical given the assumption that existing measures are generalizable across the demographically and culturally diverse U.S. population of youth. Ensuring that our measures perform equivalently across young people from different backgrounds is essential for interpreting scores and for accurate screening data and prevalence estimates of depression symptoms. As an example, the presence of DIF, uncorrected, can contribute to over-estimates of clinically elevated symptom severity in racially minoritized youth (Children’s Depression Inventory; Vaughn-Coaxum et al., 2016). Additionally, core Major Depressive Disorder symptoms such as anhedonia and psychomotor disturbances may be more difficult for female youth to endorse compared to male youth on screening measures implemented nearly universally in the U.S. pediatric care system (Patient Health Questionnaire-8; Hodgson et al., 2023).

Shifting population demographics are also relevant for the estimation of nationally representative norms and percentiles for interpreting symptom scores on questionnaire measures. Prior research in the 1990s and early 2000s generated proposed cut-off scores on the parent-report MFQ for clinically significant symptoms. In clinical samples, cut-offs of 17–21 have been suggested (Kent et al., 1997; Wood et al., 1995), whereas a normative school-based (aged 11–16) study proposed a cut-off of 20 (Cooper & Goodyer, 1993). Other researchers (i.e., Burleson Daviss et al., 2006) using a mixed clinical and community sample, reported a higher cut-off of 27 for identifying probable major depressive disorder. Without nationally representative norms and percentiles, however, it is difficult to interpret the magnitude of lower scores in community samples or to establish developmentally appropriate thresholds for screening. Further, national surveys that used self-report measures of youth symptoms over time showed that parental reports of emotional and behavioral problems remained largely stable for youth between 4 and 14 years of age between 2001 and 2019, whereas the 12-month incidence rate of major depressive episodes in 12–13 year olds increased from 3.3% in 2004 to 9.2% in 2021 (22.1% in girls and 7.8% in boys) (National Center for Health Statistics, 2020). Studies of depression symptoms specifically support homotypic continuity from early through middle childhood (preadolescence; Finsaas et al., 2018) and structural stability in the frequency and importance of symptoms endorsed (Morken et al., 2021).

Such results highlight the importance of ensuring that item characteristics for measures of youth depression symptoms are equivalent across contemporary demographic characteristics, and that nationally representative norms are available to aid in interpretation. Thus, the goals of the present study were (1) to examine item characteristics and DIF for the parent-report MFQ for school-aged and pre-adolescent children with data from an up-to-date, nationally representative sample of parents, and (2) to estimate national norms and percentiles for total scores. This analysis uses IRT models to evaluate item characteristics (item location and discrimination) and DIF across parental and child sex, and parental race and ethnicity.

## Methods

All research activities for the larger study (Lindhiem et al., 2026) were reviewed and approved by the Institutional Review Board at the [University of Pittsburgh].

### Procedure

Data collection was managed by the survey company YouGov, with all responses collected in February 2024. YouGov recruits participants through web-advertising, email, random digit dialing, postal service mailings, and contact through partner companies and organizations. They have recruited over 2 million U.S. residents to participate in their panels. Sampling for the larger study (Lindhiem et al., 2026) was constructed to match the parent-only subset of the 2022 American Community Survey (ACS), stratified for demographic representation of parents of children ages 5–12 years in the United States. The weighting of matched cases was based on propensity scores that included age, sex, racial and ethnic identity, total years of education, and geographical region of residence. YouGov sent survey invitations to 3,388 adults, and 66.7% of recipients were eligible for the study. The response rate was 57.8% and all respondents were compensated $25 for survey completion. Respondents who had more than one child in the 5–12-year-old age range were asked to report on their child with the most recent birthday. YouGov performed data quality assurances for all surveys collected, including analysis of unusual or inconsistent responses, attention checks, and identity verification (see Supplemental Material for additional detail). Following quality assurance, screen outs, incomplete responses, and responses that exceeded the quota for specific groups, a nationally-representative matched sample of 1,000 final cases was produced for analysis.

### Participants

Participants were 18 years of age or older, the biological parent or legal guardian of a child that resided with them and was 5–12 years of age, and had access to a device (e.g., computer, tablet, smartphone) with internet access for survey completion. The final sample for the larger study included 1,000 adults (57.3% female). The full sample was used to compute MFQ percentile scores and confidence intervals. The psychometric IRT analysis included the subset of adults who self-identified with the three largest racial and ethnic groups in the U.S. population (*N* = 897, 43.1% male): White or Caucasian (66.7%), Black or African American (13.4%), and Hispanic or Latine (20%). The sample sizes for adults self-identifying as Asian American and Pacific Islander, Native American and Native Hawaiian, Middle Eastern and North African, and multiracial were underpowered to include in the Differential Item Functioning analyses. Additional demographic information for the full sample and the IRT subsample are presented in Table 1.

**Table 1:** Participant Demographics Characteristics

	*Full Sample *(*N* = 1000)	*IRT Subsample* (*N* = 897)
Parent/Guardian Age	M = 40.0; SD = 7.4	M = 40.3; SD = 7.3
Parent/Guardian Assigned Sex		
Female	57.3%	56.9%
Male	42.7%	43.1%
Child Age	*M* = 8.8; *SD* = 2.2	*M* = 8.8; *SD* = 2.2
Child Assigned Sex		
Female	45.5%	44.7%
Male	54.5%	55.3%
Parent/Guardian Race		
White	76.2%	66.7%
Black or African American	13.6%	13.4%
Asian	4.8%	--
American Indian or Alaska Native	3.7%	--
Native Hawaiian or Pacific Islander	0.6%	--
Another race not listed	5.0%	--
Parent/Guardian Ethnicity		
Hispanic, Latine, or Spanish	17.9%	20%
Child Race		
White	70.0%	76.9%
Black or African American	11.7%	12.6%
Asian	3.0%	0.1%
American Indian or Alaska Native	1.3%	0.4%
Native Hawaiian or Pacific Islander	0.3%	0.3%
Another race not listed	4.3%	4.1%
Multiracial	8%	4.8%
Prefer not to say	1.4%	0.8%
Child Ethnicity		
Hispanic, Latine, or Spanish	20%	21.6%
Parent/Guardian Education		
High school or lower	29.4%	30.4%
Some college or higher	70.1%	69.2%
Prefer not to say	0.5%	0.4%
Annual Household Income		
Less than $30,000	12.3%	12.4%
$30,000 - $49,999	11.7%	11.9%
$50,000 - $74,999	17.8%	17.8%
$75,000 - $99,999	18.6%	19.2%
$100,000 and above	34.4%	34.0%
Prefer not to say	5.2%	4.7%

### Measures

Demographic questions were administered to each participating adult to solicit individual and family information including sex at birth, racial and ethnic identity, age, U.S. state of residence, marital status, employment status, number of children, and number of children in the study age range. Adults also reported on the characteristics of the target child for whom they were completing the survey, including that child’s age, sex at birth, gender identity, and racial and ethnic identity. More detailed demographic information is provided elsewhere for the full sample [publication, masked for review].

The Mood and Feelings Questionnaire (MFQ; Angold et al., 1995; Costello & Angold, 1988) was administered to all participants to report on depression symptom severity for their child. The long-form parent-report version of the MFQ includes 34 items assessing symptoms in the last 2 weeks, rated on a 3-point scale (0 = “not true”; 1 = “sometimes”; 2 = “true”). Symptoms capture the primary DSM criteria for Major Depressive Disorder, and span both affect and functioning (e.g., low mood, loss of enjoyment, appetite disturbances, sleep disturbances, fatigue, self-criticism and poor self-image, guilt, concentration difficulties, psychomotor agitation and retardation, suicidal ideation, and hopelessness about the future). Internal consistency was high in the present sample (Cronbach’s α = 0.97), and construct validity has been supported in prior studies (e.g., Jeffreys et al., 2016).

### Transparency and Openness

This study’s design and its analysis were not preregistered. Due to the data sharing policies of the lead author’s institution and the National Institutes of Health, all data and research materials are available upon request from the first author. Data were analyzed using R, version 4.5.1 (R Core Team, 2025) and the packages *mirt* and *lordif* (Chalmers, 2012; Choi et al., 2011). The R script for the data analysis is available online: https://osf.io/hz9sa.

### Data Analysis

Preliminary analyses involved exploration of the dimensionality of the MFQ items to determine whether the assumption of unidimensionality was met for fitting an IRT model. Unidimensionality was tested with three accepted methods. The first method was to examine the ratio of the first to second eigenvalues from an exploratory factor analysis (Hawes et al., 2014; Morizot et al., 2007), using a ratio ≥ 3 to support unidimensionality. The second method was computation of the Very Simple Structure criterion (VSS), which is a degradation of the factor solution, testing how well the factor matrix fits the correlation matrix. The maximum value achieved represents the ideal number of factors to extract. The third method was the Velicer Minimum Average Partial (MAP) test, which identifies the squared average partial correlations among the scale items after removing the effect of the factors.

### Item Response Theory (IRT) and Differential Item Functioning (DIF) Analyses

IRT analyses were conducted using the R Statistical Environment version 4.5.1 (R Core Team, 2025). Local independence of items was tested by examining their residual correlations after accounting for the latent trait score. We generated item characteristic curves (ICCs), item parameters, and indices of differential item functioning (DIF) for each item. We used the polytomous Graded Response Model to estimate the discrimination (α) and item location (β) parameters for each item. The discrimination (α) parameter gives the slope of the item characteristic curve (ICC) Higher α parameters indicate better discrimination. The item location parameter (β) represents the transition point, or category boundary location, between the response levels (0 to 1, and 1 to 2) of each item, and is defined as the latent trait level (theta: θ) at which a participant has a 0.50 chance of answering 0 or 1, and 1 or 2. The Graded Response Model was fit using the *mirt* R package (Chalmers, 2012) for the full dataset.

DIF analyses were performed to evaluate whether item discrimination or item location parameters differed across the three largest racial/ethnic groups in the United States (White or Caucasian, Black or African American, and Latine or Hispanic), and across parent sex at birth and child sex at birth (male and female). Significant DIF indicates that the items perform differently across the relevant categories, even when the latent trait scores for respondents are equivalent. Null findings support measurement invariance across groups. DIF analyses were performed using the *lordif* package in R (Choi et al., 2011), which implements a logistic regression approach to DIF detection. Three separate logistic regression models were compared for the full-scale score (model 1), group membership in response to the reference group (model 2), and the interaction between group membership and scale score (model 3). Chi-square values were compared across models, with significant values indicating the presence of DIF, and pseudo-R squared values were generated to estimate effect sizes, defined as the gain in log-likelihood from the explanatory variables in the model.

Variable effect size thresholds have been proposed for determining whether identified DIF is meaningful, with little consensus in the literature (Choi et al., 2011). Some of the more common thresholds include pseudo-R^2^ values > 0.13 for moderate effects (Zumbo, 1999), change in β coefficients from Model 1 to Model 2 ranging from 1% to 10% (Crane et al., 2004, 2007), and standardized impact indices computed from descriptives including item response functions (Kim & Bolt, 2007). Thus, Choi and colleagues ( 2011) proposed a Monte Carlo simulation approach that “involves generating multiple datasets (of the same dimension as the real data) under the null hypothesis (i.e., no DIF), preserving observed group differences in ability (trait level). Various magnitude measures are computed repeatedly over the simulated datasets, from which the empirical distributions are obtained.” The result is an empirical threshold for each χ^2^, β, and pseudo-R^2^ test statistic. True values exceeding Monte Carlo simulated values represent DIF that may be non-negligible and worth further investigation. Additionally, item response functions and true score functions were inspected visually and evaluated for all items where test statistics exceeded the Monte Carlo simulation threshold. To examine the potential impact of DIF on clinical interpretation of symptom severity, we identified parent-reported scores at or above the 90th percentile for MFQ raw sum scores. For all parent-reported raw sum scores meeting these criteria, we calculated which scores also met the 90th percentile criteria using the DIF-adjusted θ scores for trait estimates of symptom severity.

### Percentile Norms and Confidence Intervals

We generated percentile ranks for the raw sum scores on the MFQ, calculated following the approach recommended by Crawford et al. (2009) for handling tied scores (i.e., more than one individual indicating same raw score), based on the original method proposed by Ley (1972). Specifically, percentile rank was computed using the formula:

$$\:Percentile\:Rank=\left(\frac{m\:+\:0.5k\:}{N}\right)\times\:100$$

where *m* is the number of individuals in the normative sample scoring below a given value, k is the number of individuals with tied scores, and N is the total sample size.

To account for estimation error when applying percentile norms to the broader population, given that MFQ total scores in this sample are highly skewed and zero-inflated, 95% confidence intervals were also computed. We employed the Agresti-Coull method to calculate confidence intervals, which provides improved accuracy over the widely used Wald method, especially in large-sample contexts (Agresti & Coull, 1998; Brown et al., 2001). This method is also preferred over the Clopper-Pearson approach, which yields conservative estimates (Crawford et al., 2009). Confidence intervals were calculated using the *binom* package in R (Dorai-Raj, 2006).

## Results

**Fig. 1:**
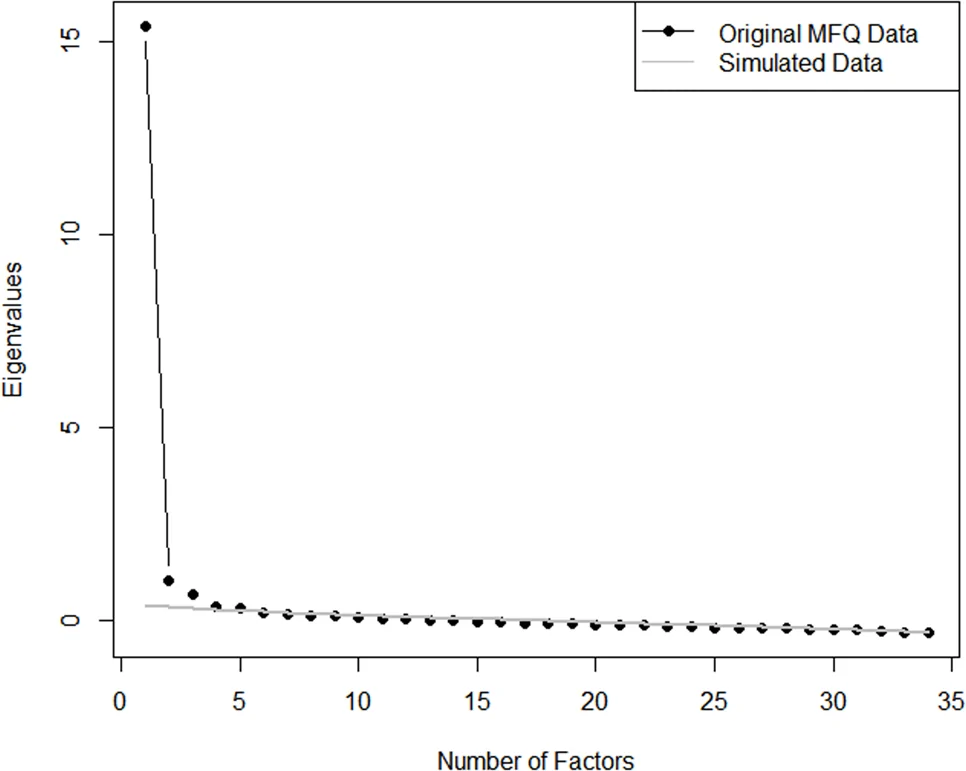
Scree Plot. Note. Eigenvalues extracted from Exploratory Factor Analysis to examine unidimensionality of the parent-report MFQ. A Parallel Analysis was performed to empirically simulate eigenvalues that would be produced by chance. True eigenvalues above the solid line for simulated data are unlikely to have been produced by chance

**Fig. 2:**
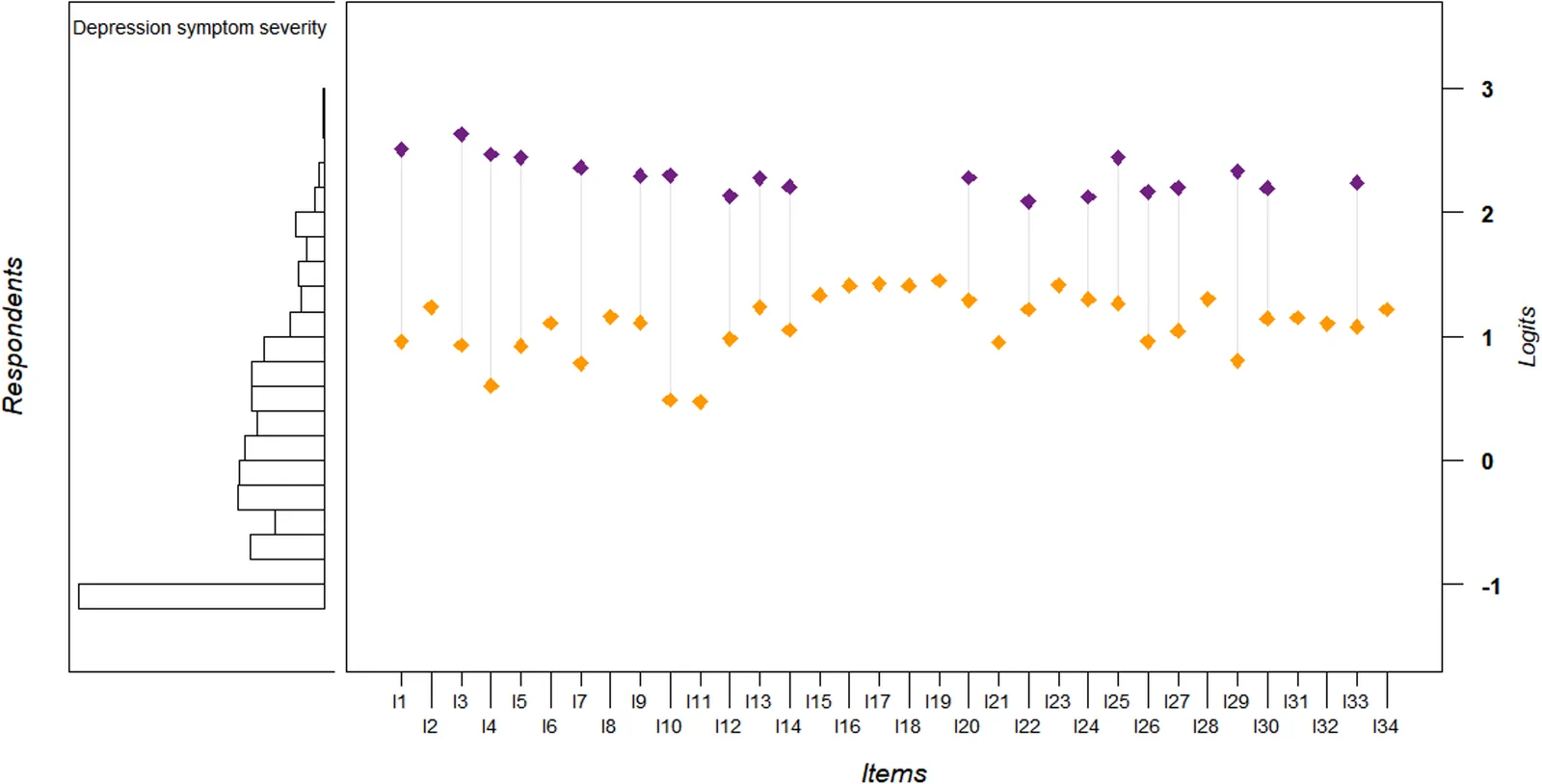
Person-Item Map of Depression Severity Theta Distributions and Item Location Thresholds. Note. The left respondent panel represents the frequency distribution of trait depression symptom severity scores (θ), mapped to item location parameters (thresholds for item category threshold transitions) for each individual MFQ item. The lower diamonds represent the item location threshold between a response of 0 and 1, and the upper diamonds represent the item location thresholds between a response of 1 and 2. Items with only a single diamond do not have an estimate location threshold for a response of 1 and 2 due to low frequency of endorsement of the highest response category

**Fig. 3:**
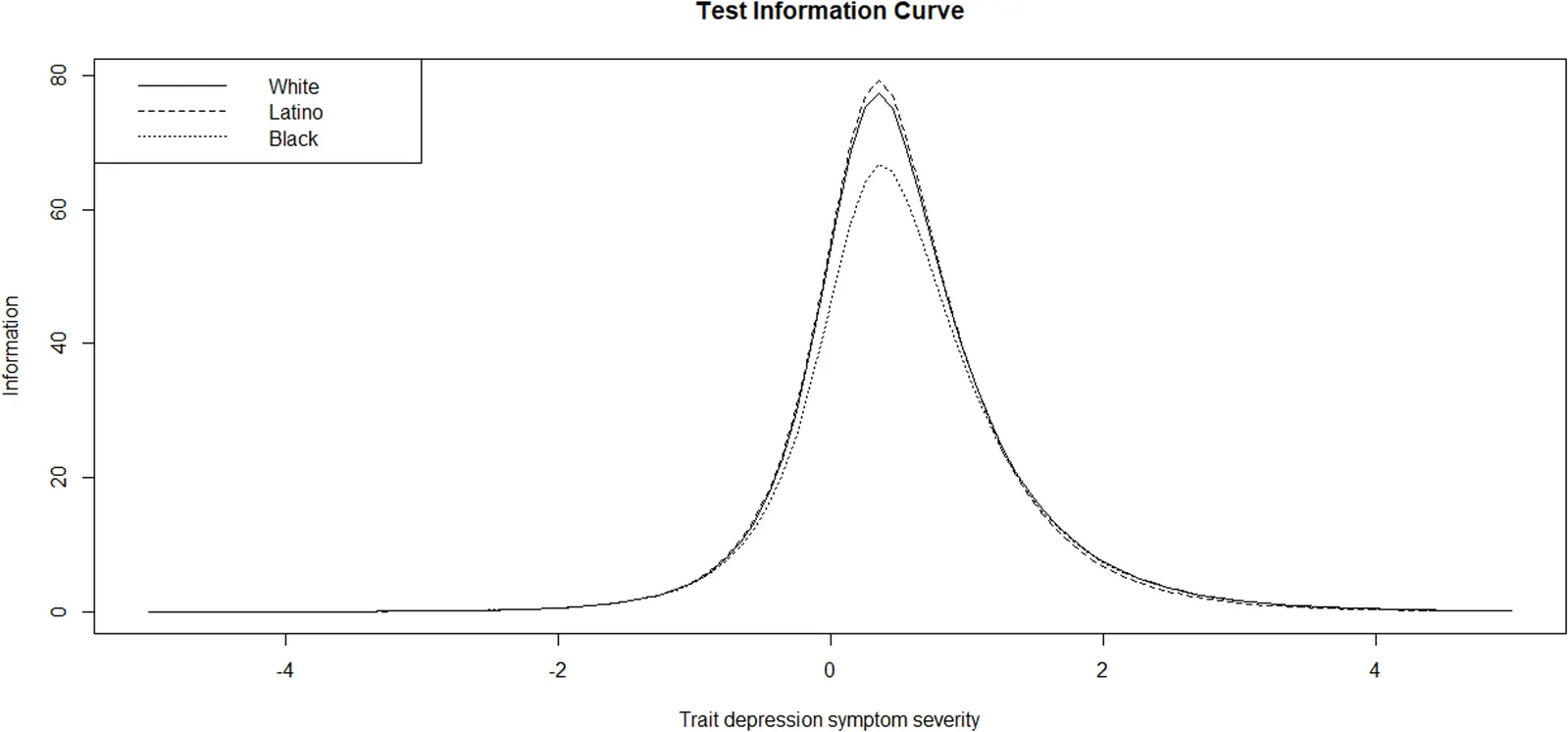
Test Information Curve. Note. Total test information plotted by parental race and ethnicity across latent trait scores (θ) of depression symptom severity estimated from the Graded Response Model

Unidimensionality was supported for the full MFQ parent-report scale. For the full sample, the results of the parallel analysis are presented in Fig. 1 and the ratio of the first to second eigenvalue was 14:1. The goodness-of-fit statistics also supported unidimensionality (RMSR = 0.05; RMSEA = 0.07), and the VSS complexity 1 reached a maximum value of 0.95 with one factor. The IRT graded response model showed an acceptable fit to data for all respondents (RMSEA = 0.04, SRMSR = 0.04, TLI = 0.99, CFI = 0.99). Item parameters extracted from the GRM for the full sample revealed that the item discrimination parameter across all 34 items ranged from α = 1.28 to 3.99, suggesting that all items exceed a conventional threshold for “good” discrimination (0.80; De Ayala, 2009) and that the location parameters demonstrate sufficient range, documenting that the underlying latent construct of depression symptoms is broad enough to effectively discriminate between individuals across the latent trait (see Fig. 2). For the full sample, Fig. 3 illustrates the total information curve for the range of symptom severity captured by the MFQ for each racial and ethnic subgroup of parents.

Results of the DIF analysis varied by child sex and parent sex and race and ethnicity. No items were flagged for DIF across parent sex, supporting measurement invariance for this variable. One item was flagged for DIF across child sex, and nine items were flagged for DIF across parent race and ethnicity. Empirical cutoffs for the χ^2^, β, and pseudo-R^2^ test statistics for uniform and non-uniform DIF were based on Monte Carlo simulated datasets, and none of the items flagged for DIF by child sex or parent race or ethnicity exceeded the thresholds for all three test statistics for uniform or non-uniform DIF, suggesting that these effects were modest and unlikely to be of clinical significance. Item discrimination and category location estimates are presented for all items in Table 2; for the 10 items with likely non-significant DIF, discrimination and location parameters are recalculated to adjust for DIF by group and presented separately for each subgroup for illustrative purposes.

**Table 2:** IRT Item Parameters for Long-Form Parent Report MFQ

Item	Full sample		
	a	cb1	cb2
I1. Sadness*	2.37	0.96	2.51
I2. Lack of enjoyment*	2.68	1.23	*NA*
I3. Low appetite	1.58	0.93	2.63
I4. Increased appetite	1.25	0.60	2.47
I5. Fatigue*	2.14	0.92	2.44
I6. Psychomotor slowing	3.03	1.11	*NA*
I7. Restlessness*	1.80	0.78	2.36
I8. Worthlessness*	3.98	1.16	*NA*
I9. Self-blame	2.31	1.11	2.29
I10. Indecision	1.58	0.49	2.30
I11. Irritability	1.59	0.47	*NA*
I12. Talking less	2.69	0.98	2.13
I13. Slowed speech	2.98	1.23	2.28
I14. Crying*	2.12	1.05	2.21
I15. Hopeless about the future	3.29	1.33	*NA*
I16. Thoughts of life not worth living	4.02	1.41	*NA*
I17. Thoughts of death	2.87	1.43	*NA*
I18. Thoughts of family being better off without them	3.91	1.41	*NA*
I19. Thoughts of suicide	4.86	1.45	*NA*
I20. Social withdrawal	2.77	1.29	2.28
I21. Difficulty concentrating*	2.36	0.95	*NA*
I22. Thoughts of bad things happening to them	3.51	1.22	2.09
I23. Self-hatred*	4.19	1.41	*NA*
I24. Thoughts of self as bad person*	3.94	1.30	2.12
I25. Dislikes appearance	2.63	1.27	2.44
I26. Worries about aches and pains	2.16	0.96	2.17
I27. Loneliness*	2.55	1.04	2.20
I28. Feels unloved*	3.22	1.30	*NA*
I29. Lack of school enjoyment	1.97	0.80	2.33
I30. Self-deprecation (not as good as other kids)*	3.02	1.14	2.19
I31. Feels that they do everything wrong*	3.29	1.15	*NA*
I32. Difficulty sleeping (quality or duration)	1.80	1.10	*NA*
I33. Hypersomnia (sleeps more than usual)	2.17	1.08	2.24
I34. Low positive affect, reduced reward sensitivity	3.30	1.22	*NA*

*=item also included in the Short-Form Mood and Feelings Questionnaire (SF-MFQ). *a*= discrimination parameter, *cb1* = category location threshold between response categories 0 and 1, *cb2* = category location threshold between response categories 1 and 2, *NA* = no estimate of category location threshold (*cb2*) due to low frequency of endorsement of response category 2. Item parameters provided for the full IRT sample, including all racial, ethnic, and sex

**Fig. 4:**
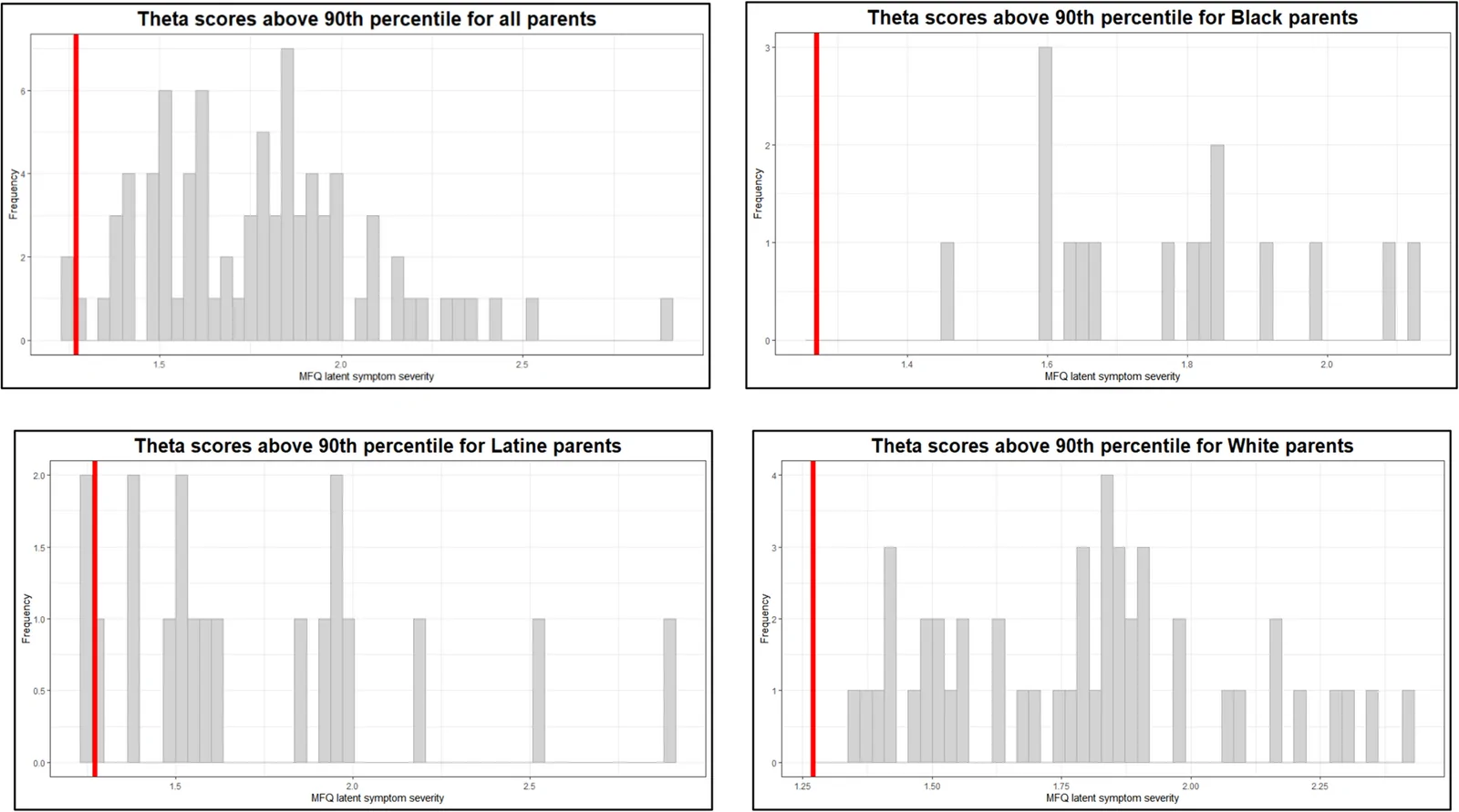
Concordance of MFQ Raw Scores and IRT Theta Scores. Note. The distribution of respondents’ MFQ raw sum scores at or above the 90th percentile (23) that also fall at or above the 90th percentile of IRT derived theta scores for all participants and by each racial and ethnic subgroup. The solid vertical line in each histogram represented the 90th percentile cutoff for the IRT theta scores. All cases to the right of the vertical line are classified by both scoring methods as 90th percentile or above. Cases to the left of the solid vertical line represent discordance between scoring methods (cases classified as 90th percentile by the MFQ raw sum score, but falling below the 90th percentile based on IRT theta scores)

Overall percentiles rankings and corresponding confidence intervals were calculated for the total raw sum scores of the MFQ scale (Table 3). Additional percentiles and confidence intervals, stratified by binned child age group (5–6 years old, 7–8 years old, 9–10 years old, and 11–12 years old), are provided in the Supporting Information (Table S1). Further, percentile rankings were compared across IRT-derived latent θ scores and traditional raw sum scores across items to evaluate agreement between scoring methods for identifying clinically elevated symptom levels. The 90th percentile is commonly used to identify clinically elevated symptoms across measures (Craighead et al., 1998), and as seen in Table 3, the 90th percentile corresponds to a raw MFQ sum scores of 23, and a θ score of 1.27. Frequency distributions of θ scores for participants with raw sum scores ≥ 23 were examined across each subgroup for parent racial and ethnic identity (Fig. 4). All scores among Black and White parents meeting the 90th percentile cutoff for MFQ raw sum scores also met the 90th percentile cutoff for θ scores. For Latine and Hispanic parents, 10% of scores that met the 90th percentile cutoff for MFQ raw scores were just below the θ cutoff. The overall results suggest that the θ scores may be slightly more conservative, though the traditional raw sum scores largely perform well.

**Table 3:** Percentile Rank and 95% Confidence Intervals of MFQ Total Raw Scores in Normative Sample

Raw MFQ sum Score	Percentile	95% Confidence Interval
0	11	10–13
1	29	26–32
2	40	37–43
3	48	45–51
4	57	53–59
5	61	58–64
6	65	62–68
7	69	66–72
8	72	69–75
9	75	72–78
10	78	75–80
11	80	77–82
12	81	78–83
13	82	80–85
14	84	81–86
15	85	83–87
16	86	84–88
17	87	85–89
18	88	86–90
19	89	87–91
20	90	88–91
21	90	88–92
22	91	89–92
23	91	89–93
24	92	90–93
25	92	90–94
26	92	90–94
27	93	91–94
28	93	91–94
29	93	92–95
30	94	92–95
31	94	92–95
32	94	93–95
33	94	93–96
34	95	94–96
35	96	94–97
36	96	95–97
37	96	95–97
38	97	95–98
39	97	96–98
40	97	96–98
41	97	96–98
42	98	97–99
43	98	97–99
44	98	97–99
45	99	98–99
46	99	98–99
47	99	98–100
48	99	99–100
49	99	99–100
50	>99	99–100
51	>99	99–100
52	>99	99–100
53	>99	99–100
54	>99	99–100
55	>99	99–100
56	>99	99–100
57	>99	99–100
58	>99	99–100
59	>99	99–100
60	>99	99–100
61	>99	99–100
62	>99	99–100
63	>99	99–100
64	>99	99–100
65	>99	99–100
66	>99	99–100

## Discussion

The quality of our assessments of depression symptoms in youth, both in clinical settings and research, depend on the quality of our measurement tools (Achenbach, 2006). Advancing scientific research on the etiology and treatment of youth depression and increasing the use of measurement-based care necessitates that our measures adequately capture the range of depression symptom severity and that they do so equivalently across different groups of individuals to ensure that scores have the same meaning and interpretation. The goal of the present analysis was to evaluate the MFQ item characteristics, test for item-level DIF (sex, race, ethnicity), and provides norms and percentiles for parent-report MFQ sum scores based on a sample weighted to represent the U.S. population of parents of children ages 5–12 years. The present study extends prior psychometric assessments of the parent-report MFQ with an IRT approach and percentile rank calculations with confidence intervals. Study results indicate that the parent-report MFQ item location parameters capture a range of latent depression symptom severity, and that all 34 long-form items discriminate sufficiently between individuals at different levels of latent symptom severity. The DIF analysis also supported equivalent item characteristics, and thus interpretation of total scores and individual symptom endorsements, across the largest racial and ethnic groups in the U.S., and by sex.

Overall, study results for the parent-report MFQ items are consistent with prior IRT research on the child self-report version, supporting equivalent item characteristics across youth racial and ethnic identity (Banh et al., 2012; Schlechter et al., 2023). Results are also consistent with prior classical test theory studies using confirmatory factor analytic methods and finding support for metric and scalar invariance on the parent report version of the MFQ (Jeffreys et al., 2016; Noureddine et al., 2025). Prior psychometric studies of the parent-report version of the MFQ have also focused largely on adolescence (Cooper & Goodyer, 1993; Kent et al., 1997; Wood et al., 1995), which is reasonable considering the spike in prevalence rates of youth depression during that developmental stage. However, this leaves measures of symptoms in younger children underexplored psychometrically. The present study addresses gaps in item-level measurement evaluation using an IRT framework to maximize the clinically relevant information available for the parent-report MFQ in younger children (e.g., ability of items to distinguish between respondents across the continuum of latent symptom severity).

The discrimination parameters of the items on the long-form parent-report MFQ were uniformly high, and all above the minimum recommended threshold. This finding indicates that items adequately distinguish between individuals at differing levels of symptom severity. The results of the item-person map also illustrate variability in item location (or category location boundary) parameters. Still, item locations are shifted somewhat toward higher latent symptom severity. Due to the high sensitivity of the DIF detection methods, the Monte Carlo simulation method was used to determine whether the effect sizes were likely to be meaningful. All effect sizes were small, and there were no items flagged for DIF that met the Monte Carlo simulation threshold for all three of the statistics evaluated: chi-square tests, change in Beta from Model 1 to Model 2, and pseudo-R squared values for each model comparison. As such, there were no items suggested for exclusion, a conclusion that supports equivalent interpretation of scores and equivalent normative cutoffs across race, ethnicity, and sex when using the MFQ for research or clinical purposes. It is also important to note that while DIF-detection provides item-level estimates for the MFQ, the lack of DIF does not indicate a lack of differences in symptom severity across demographic characteristics. While not a focus of the current analysis, there may be meaningful differences in latent symptom severity (theta scores) across youth of various ages, racial and ethnic identities, socioeconomic status, and sex and gender identity. The results of this study are focused solely on the equivalence of item characteristics, and not on group differences in depression symptoms or presentation.

The present study also provides normative scores based on a nationally representative sample of U.S. parents of pre-adolescent aged youth (ages 5–12). Prior research on the MFQ has been limited to clinical cutoff scores suggested for detecting depressive disorders, and there has been a lack of data on how scores are distributed in the population (Cooper & Goodyer, 1993; Kent et al., 1997; Wood et al., 1995). Results suggest that the 90th percentile cutoff score (23 in the current study) is consistent with previous studies with older youth or clinically referred samples, where proposed thresholds in prior studies range from 17 to 27 depending on sample composition and intended use (e.g., Burleson Daviss et al., 2006; Cooper & Goodyer, 1993; Kent et al., 1997; Wood et al., 1995). Cutoffs at the higher end of the range, and above our cutoff of 23, are unsurprising in clinical contexts where symptom severity is elevated and the focus is often on differential diagnosis. Notably, the 85th percentile score (15 in the current study) which is used for screening on similar child and parent-report measures like the CDI-2, is lower than the aforementioned cutoffs recommended for clinically elevated symptoms in research on the Mood and Feelings Questionnaire (MFQ).

The lower, 85th percentile threshold may reflect elevations relative to the general population, where absolute symptom levels are typically milder. Such a threshold may increase sensitivity and enhance the utility of the MFQ as a screening instrument in community, school, or primary care settings. Prior work on the parent-report MFQ highlights trade-offs between sensitivity and specificity across cutoff scores. Lower thresholds prioritize sensitivity (e.g., 0.88 sensitivity, 0.70 specificity), supporting use in screening contexts, whereas higher thresholds improve specificity (e.g., 0.85 specificity, 0.61 sensitivity at a cutoff of 27), making them more appropriate for diagnostic decision-making (Costello & Angold, 1988; Burleson Daviss et al., 2006). Intermediate cutoff scores (approximately 21–25) yield more variable and often modest balance between sensitivity (0.63–0.67) and specificity (0.61–0.80), depending on the sample (Wood et al., 1995; Kent et al., 1997). Notably, more stringent thresholds can achieve very high specificity (up to 1.00) but at the cost of substantially reduced sensitivity (e.g., 0.34), underscoring the difficulty of maximizing both metrics simultaneously (Kent et al., 1997). Consistent with this literature, the present findings suggest that a lower cutoff (15) may be appropriate for screening for elevated depressive symptoms or symptoms of MDD. These types of screening procedures are often used clinically in pediatric primary care or school settings. A higher threshold (23) better aligns with prior cutoffs derived from clinical samples that may be more appropriate for identifying probable depressive disorders. This cutoff may be particularly useful when administering the MFQ as an intake or treatment outcome measure, for example, and in clinical trials research on youth depression. Thus, the current study expands the literature by providing developmentally appropriate, population-based guidance for MFQ score interpretation in younger children.

### Constraints on Generality

ThCg and sampling procedures, which ensured that the findings are representative of parents of children in the United States. In addition, the sample size was substantially larger than in prior psychometric studies, providing stable estimates of percentiles and suggested thresholds for interpreting sum scores. Importantly, this study focused on a younger age group (children 5–12 years), for whom normative data and empirically derived thresholds are largely lacking.

The study results should also be interpreted in the context of the following limitations. Although the study sample was representative regarding parent race and ethnicity, the overall sample size was not sufficient for testing DIF across all participants’ racial and ethnic identities. Individuals identifying with the Asian diaspora are one of the largest growing groups in the U.S. youth population; however, the number of participants in this sample identifying from the Asian diaspora was not large enough to include these individuals in the DIF analysis. Also, because the sample was designed to be nationally representative, certain demographic subgroups (e.g., Black and Latine parents) were represented in smaller numbers relative to White parents. As a result, there was less variability at the most severe end of the symptom continuum (90th percentile) within these racial and ethnic groups. Additionally, it is important to restrict the norms and percentile cutoffs to the long-form MFQ. Although early validity studies suggest that the Short Form (SMFQ, parent-report) demonstrates sufficient sensitivity and specificity to distinguish youth with and without depressive disorders in ages 7 years and older (Burleson Daviss et al., 2006; Thapar & McGuffin, 1998), our aim was to evaluate item-level characteristics and DIF for all possible MFQ items. Thus, the estimation of norms and percentiles, and recommended cutoff scores for the 85th and 90th percentile, are specific to the long-form MFQ .

## Conclusions and Future Directions

The present findings support the equivalence of the item-characteristics of the Mood and Feelings Questionnaire (MFQ) across diverse demographic groups, with comparable reporting by mothers and fathers and by parents identifying with the largest racial and ethnic groups in the U.S. This study addresses a gap in the literature, which focuses on psychometrics for measuring symptom severity in older youth and clinically referred samples, by establishing developmentally appropriate parent-reported score thresholds based on percentiles in a large, nationally representative sample of parents of younger children. Critically, the norms estimated in the current study are applicable only to the parent-report version of the MFQ. Norms and cutoff score recommendations are not intended to translate to child self-report scores from children ages 5–12 without empirical testing, which is an important next step for this widely used measure. Future studies should also consider whether other important patient characteristics may influence item-level measurement equivalence and warrant DIF evaluation (e.g., parental psychopathology, socioeconomic indicators, more nuanced cultural factors beyond race and ethnicity). The current study contributes (1) item-level psychometric performance, and (2) norms for total scores, to the evidence base for the parent-report MFQ. Results extend prior psychometric work and extend the availability of suggested cutoff scores to younger children. Together with prior psychometric research, this study supports the utility of the parent-report MFQ for depressive symptom screening and identifying clinically elevated symptom levels in demographically diverse youth populations.

## Supplementary Information

Supplementary Material 1.

## Data Availability

Data is available upon request. Due to the data sharing policies of the lead author’s institution and the National Institutes of Health, a data use agreement is required with the lead author’s institution.
