# Supplementary material for: National Norms and Differential Item Functioning Tests of the Parent-Report MFQ for Children Ages 5–12
Source: J Psychopathol Behav Assess. 2026 Jul 17;48(3):41. doi: 10.1007/s10862-026-10300-9 (PMC13379435; doi:10.1007/s10862-026-10300-9)
Supplement: Supplementary file 1 — Supplementary Material 1. [file 10862_2026_10300_MOESM1_ESM.docx]

**Supplemental Material**

**YouGov data assurance methodology and survey response statistics**. YouGov provides detailed information on all aspects of survey methodology, including data assurance, here: <https://yougov.com/en-us/about/methodology>. Several procedures are implemented to ensure quality, representative, responses are obtained and analyzed. First, YouGov verifies panelists’ (potential participants) identities via IP address checks and email address verification, as well as metadata from panelists’ devices and location. YouGov also uses data about panelists to perform what they call a “response quality survey”, comparing actual survey responses to predicted or known information about the individual. Additionally, YouGov embeds quality control checks within each survey to identify any response issues related to the following: response latency that is too fast, inconsistent responses, or repeated endorsement of the same answer to consecutive questions. Responses that do not pass quality control checks are excluded from the final sample. YouGov continues to recruit and obtain responses until the target sample is reached with responses that pass quality assurance procedures. The target sample for the larger study was 1,000 adults, nationally-representative of parents of 5-12 year old children in the U.S. YouGov sent 3,388 invites to potential participants. The eligibility rate was 66.70%, and the response rate was 57.80%. After removal of incomplete responses, screen outs, and responses for groups where the quota was full, YouGov received 1,102 responses that passed all quality control and data assurance checks. The sample was matched down to 1,000 participants based on gender, age, race, and education for population-statistics of U.S. parents of 5-12 year old children. Sample weights were based on propensity scores using the 2022 American Community Survey.

**Table S1.** Percentile Rank and 95% Confidence Intervals (95% CI) of MFQ Total Raw Scores in Normative Sample Binned by Age Groups

| **Raw MFQ Score** | **5-6 years** | | **7-8 years** | | **9-10 years** | | **11-12 years** | |
| --- | --- | --- | --- | --- | --- | --- | --- | --- |
|  | **Percentile** | **95% CI** | **Percentile** | **95% CI** | **Percentile** | **95% CI** | **Percentile** | **95% CI** |
| 0 | 10 | 7–15 | 10 | 7–15 | 12 | 8–16 | 13 | 9–17 |
| 1 | 27 | 21–33 | 28 | 22–34 | 29 | 24–34 | 32 | 27–37 |
| 2 | 38 | 32–45 | 39 | 33–45 | 39 | 33–45 | 43 | 37–49 |
| 3 | 47 | 40–54 | 46 | 39–52 | 48 | 43–54 | 51 | 45–57 |
| 4 | 54 | 48–61 | 52 | 46–59 | 57 | 51–63 | 58 | 52–63 |
| 5 | 61 | 54–67 | 57 | 50–63 | 62 | 57–68 | 63 | 57–68 |
| 6 | 66 | 59–72 | 61 | 55–67 | 66 | 60–71 | 67 | 62–73 |
| 7 | 70 | 63–75 | 66 | 60–72 | 69 | 63–74 | 71 | 65–76 |
| 8 | 72 | 65–78 | 71 | 64–76 | 71 | 66–76 | 74 | 69–79 |
| 9 | 75 | 69–80 | 75 | 68–80 | 74 | 69–79 | 77 | 72–82 |
| 10 | 78 | 72–84 | 77 | 72–82 | 77 | 72–81 | 79 | 74–84 |
| 11 | 80 | 74–85 | 79 | 73–84 | 79 | 74–83 | 80 | 75–85 |
| 12 | 81 | 75–86 | 81 | 75–85 | 81 | 76–85 | 81 | 76–85 |
| 13 | 82 | 76–87 | 82 | 77–87 | 83 | 78–87 | 82 | 77–86 |
| 14 | 84 | 78–88 | 83 | 78–88 | 84 | 80–88 | 84 | 80–88 |
| 15 | 85 | 79–89 | 84 | 79–88 | 86 | 81–89 | 86 | 82–90 |
| 16 | 86 | 80–90 | 86 | 80–90 | 87 | 82–90 | 88 | 83–91 |
| 17 | 86 | 81–90 | 87 | 82–91 | 87 | 83–91 | 89 | 84–92 |
| 18 | 87 | 82–91 | 88 | 83–91 | 88 | 84–92 | 89 | 85–92 |
| 19 | 88 | 83–92 | 88 | 84–92 | 89 | 85–92 | 90 | 85–93 |
| 20 | 89 | 84–92 | 89 | 84–93 | 90 | 86–93 | 91 | 87–94 |
| 21 | 89 | 84–92 | 89 | 85–93 | 91 | 87–94 | 91 | 87–94 |
| 22 | 90 | 85–93 | 89 | 85–93 | 91 | 88–94 | 91 | 88–94 |
| 23 | 92 | 87–95 | 90 | 85–93 | 92 | 88–95 | 92 | 88–94 |
| 24 | 93 | 88–96 | 90 | 86–93 | 92 | 88–95 | 92 | 88–95 |
| 25 | 93 | 89–96 | 90 | 86–94 | 92 | 89–95 | 92 | 88–95 |
| 26 | 94 | 89–96 | 91 | 86–94 | 92 | 89–95 | 92 | 89–95 |
| 27 | 94 | 90–97 | 91 | 87–94 | 93 | 89–95 | 93 | 89–95 |
| 28 | 94 | 90–97 | 92 | 88–95 | 93 | 89–95 | 93 | 89–95 |
| 29 | 95 | 91–97 | 92 | 88–95 | 93 | 89–96 | 93 | 90–96 |
| 30 | 95 | 91–97 | 93 | 89–95 | 93 | 90–96 | 94 | 90–96 |
| 31 | 95 | 91–97 | 93 | 89–96 | 94 | 90–96 | 94 | 91–96 |
| 32 | 95 | 91–98 | 93 | 89–96 | 94 | 90–96 | 94 | 91–97 |
| 33 | 96 | 92–98 | 93 | 89–96 | 94 | 91–96 | 94 | 91–97 |
| 34 | 96 | 92–98 | 94 | 90–97 | 95 | 92–97 | 95 | 92–97 |
| 35 | 96 | 92–98 | 95 | 91–97 | 96 | 93–98 | 96 | 93–98 |
| 36 | 96 | 93–98 | 95 | 92–97 | 96 | 93–98 | 96 | 93–98 |
| 37 | 97 | 93–99 | 96 | 92–98 | 96 | 93–98 | 97 | 94–98 |
| 38 | 97 | 94–99 | 96 | 92–98 | 97 | 94–98 | 97 | 94–99 |
| 39 | 97 | 94–99 | 96 | 92–98 | 97 | 95–98 | 98 | 95–99 |
| 40 | 97 | 94–99 | 96 | 93–98 | 98 | 99–99 | 98 | 96–99 |
| 41 | 97 | 94–99 | 96 | 93–98 | 98 | 95–99 | 98 | 96–99 |
| 42 | 98 | 94–99 | 97 | 94–99 | 98 | 95–99 | 98 | 96–100 |
| 43 | 98 | 94–99 | 98 | 95–99 | 98 | 96–99 | 99 | 96–100 |
| 44 | 98 | 95–99 | 98 | 95–99 | 98 | 96–100 | 99 | 97–100 |
| 45 | 98 | 95–99 | 99 | 96–100 | 99 | 97–100 | 99 | 97–100 |
| 46 | 99 | 96–100 | 99 | 96–100 | 99 | 97–100 | 99 | 97–100 |
| 47 | 99 | 96–100 | 99 | 97–100 | 99 | 97–100 | 99 | 97–100 |
| 48 | 99 | 96–100 | > 99 | 97–100 | 99 | 97–100 | 99 | 97–100 |
| 49 | 99 | 96–100 | > 99 | 97–100 | 99 | 97–100 | > 99 | 98–100 |
| 50 | 99 | 96–100 | > 99 | 98–100 | > 99 | 97–100 | > 99 | 98–100 |
| 51 | 99 | 97–100 | > 99 | 98–100 | > 99 | 98–100 | > 99 | 98–100 |
| 52 | > 99 | 97–100 | > 99 | 98–100 | > 99 | 98–100 | > 99 | 98–100 |
| 53 | > 99 | 97–100 | > 99 | 98–100 | > 99 | 98–100 | > 99 | 98–100 |
| 54 | > 99 | 98–100 | > 99 | 98–100 | > 99 | 98–100 | > 99 | 98–100 |
| 55 | > 99 | 98–100 | > 99 | 98–100 | > 99 | 98–100 | > 99 | 98–100 |
| 56 | > 99 | 98–100 | > 99 | 98–100 | > 99 | 98–100 | > 99 | 98–100 |
| 57 | > 99 | 98–100 | > 99 | 98–100 | > 99 | 98–100 | > 99 | 98–100 |
| 58 | > 99 | 98–100 | > 99 | 98–100 | > 99 | 98–100 | > 99 | 98–100 |
| 59 | > 99 | 98–100 | > 99 | 98–100 | > 99 | 98–100 | > 99 | 98–100 |
| 60 | > 99 | 98–100 | > 99 | 98–100 | > 99 | 98–100 | > 99 | 98–100 |
| 61 | > 99 | 98–100 | > 99 | 98–100 | > 99 | 98–100 | > 99 | 98–100 |
| 62 | > 99 | 98–100 | > 99 | 98–100 | > 99 | 98–100 | > 99 | 98–100 |
| 63 | > 99 | 98–100 | > 99 | 98–100 | > 99 | 98–100 | > 99 | 98–100 |
| 64 | > 99 | 98–100 | > 99 | 98–100 | > 99 | 98–100 | > 99 | 98–100 |
| 65 | > 99 | 98–100 | > 99 | 98–100 | > 99 | 98–100 | > 99 | 98–100 |
| 66 | > 99 | 98–100 | > 99 | 98–100 | > 99 | 98–100 | > 99 | 98–100 |
